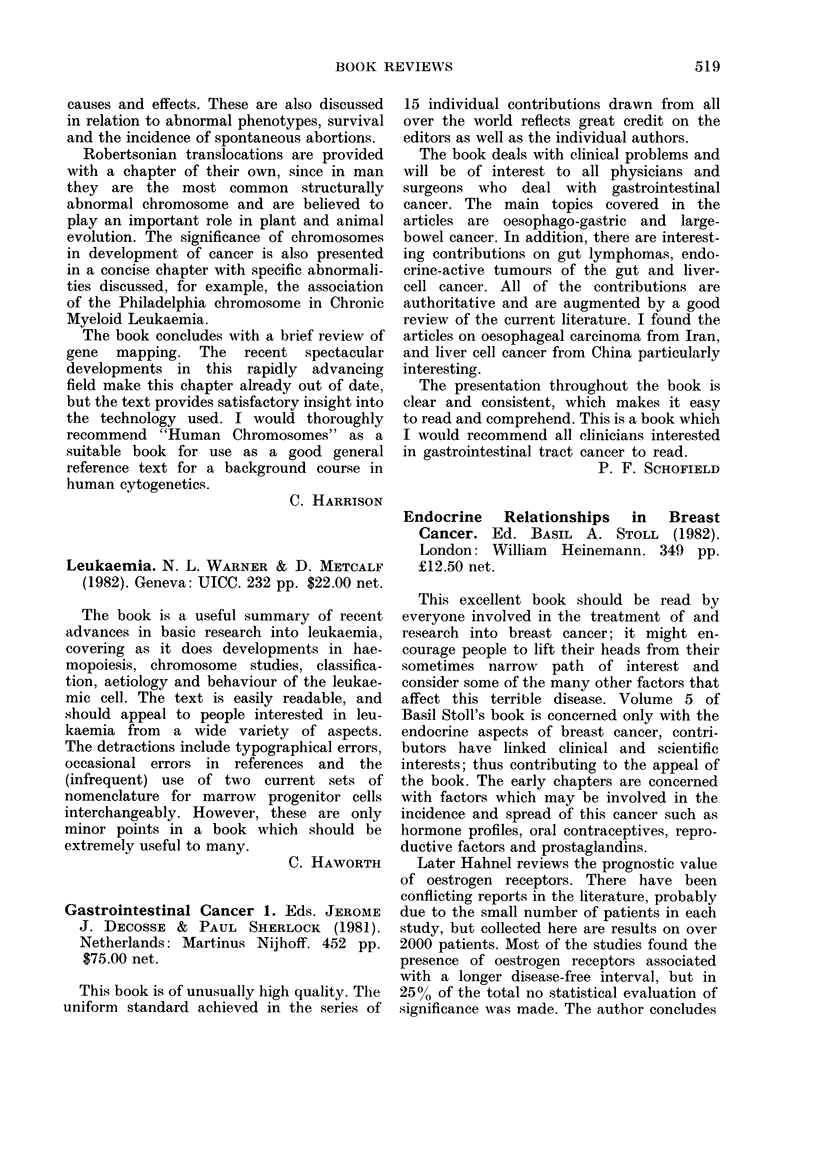# Leukaemia

**Published:** 1982-09

**Authors:** C. Haworth


**Leukaemia.** N. L. WARNER & D. METCALF (1982). Geneva: UICC. 232 pp. $22.00 net.

The book is a useful summary of recent advances in basic research into leukaemia, covering as it does developments in haemopoiesis, chromosome studies, classification, aetiology and behaviour of the leukaemic cell. The text is easily readable, and should appeal to people interested in leukaemia from a wide variety of aspects. The detractions include typographical errors, occasional errors in references and the (infrequent) use of two current sets of nomenclature for marrow progenitor cells interchangeably. However, these are only minor points in a book which should be extremely useful to many.

C. HAWORTH